# Risk factors associated with poor prognosis after endoscopic treatment of clinical T1a esophageal cancer

**DOI:** 10.1007/s00464-025-11880-5

**Published:** 2025-07-08

**Authors:** Aria Bassiri, Christina S. Boutros, Caroline Pennacchio, Amitabh Chak, Amit Bhatt, Philip A. Linden, Christopher W. Towe

**Affiliations:** 1 Division of Thoracic and Esophageal Surgery, Department of Surgery, University Hospitals Cleveland Medical Center, 11100 Euclid Avenue, Cleveland, OH 44106 USA; 2 Case Western Reserve University School of Medicine, 9501 Euclid Ave, Cleveland, OH 44106 USA; 3 Department of Gastroenterology, University Hospitals Cleveland Medical Center, 11100 Euclid Avenue, Cleveland, OH 44106 USA; 4 Department of Gastroenterology, Hepatology & Nutrition, Cleveland Clinic Foundation, Main Campus, 9500 Euclid Avenue, Cleveland, OH 44195 USA

**Keywords:** Endoscopic resection, Esophageal carcinoma, T1a

## Abstract

**Background:**

Increasingly, endoscopic techniques such as Endoscopic Mucosal Resection and Endoscopic Submucosal Dissection provide less invasive treatment for early-stage esophageal cancer. However, factors that affect survival after endoscopic resection of esophageal cancer (EREC) are poorly defined. This study aims to build a risk model for patients undergoing EREC, identifying the impact of pathologic risk factors on survival.

**Methods:**

This retrospective study utilized the National Cancer Database to analyze survival rates of patients with clinically staged T1aN0M0 esophageal cancer who received EREC between 2004 and 2019. Patients treated with chemotherapy/radiation before EREC or surgery after EREC were excluded. The primary outcome was overall survival. Demographic and tumor characteristics were evaluated for their impact on survival. These factors were used in a multivariable analysis to create a risk score, and survival rates were compared across risk scores.

**Results:**

The study analyzed 2169 esophageal cancer patients who underwent EREC. Factors such as age, comorbidity index, and tumor grade were associated with survival. A risk score was developed, which included staging post-EREC, margin status, presence of lymphovascular invasion, histology, and pathologic grade assessment. Increasing risk score was associated with increased risk of death as both a continuous and categorical variable.

**Conclusions:**

This study evaluated factors affecting survival after EREC. This risk score could be used to identify patients at higher risk of death, thus aiding in patient counseling and treatment planning. Further validation using prospective data are recommended.

Although esophagectomy has been historically offered to patients with early-stage esophageal cancer, endoscopic techniques including endoscopic mucosal resection (EMR) and endoscopic submucosal dissection (ESD) have created less invasive approaches for treating patients with early esophageal cancer [1]. The 2024 National Comprehensive Cancer Network (NCCN) guidelines suggest that endoscopic resection of esophageal cancer (EREC) is the “preferred” strategy for T1a disease [2]. Endoscopic eradication therapy (EET) is recommended for T1a esophageal adenocarcinoma (EAC) and an option for low risk (depth of invasion < 500 microns, well or moderately differentiated, no lymphovascular invasion (LVI)) T1b EAC, in the guidelines and clinical practice updates from the American College of Gastroenterology, American Gastroenterological Association, and American Society for Gastrointestinal Endoscopy [3–5].

While many patients with early-stage esophageal cancer are effectively treated by EREC, some patients will have pathologic features that increase the risk of recurrence, poor prognosis, and death [6]. Additionally, while many patients with “high risk” features are offered esophagectomy to reduce the risk of recurrence, for patients unwilling or unable to receive esophagectomy, endoscopic treatment may be the *only* treatment they receive for their cancer [7].

Several studies have attempted to determine which factors are associated with poor prognosis after EREC. Increased tumor size, poor differentiation, submucosal invasion, and LVI were more likely to have lymph node metastasis with LVI the strongest predictor of lymph node metastasis in patients undergoing esophagectomy for treatment of T1 esophageal adenocarcinoma [8]. Patients undergoing ESD resection for pathologically staged T1b EC who are unable to undergo surgery have recurrence rates of 20% and should be monitored closely [9]. While some risk factors for poor prognosis after EREC have been explored, these investigations have been limited in size and have not fully assessed the impact of demographic and pathologic features on recurrence and survival. Consequently, the influence of clinical and demographic features associated with poor prognosis after EREC remains unclear.

Given the paucity of data about factors affecting survival after EREC, we sought to create a risk model for patients receiving EREC to determine the relative impact of clinical and demographic risk factors on overall survival. The purpose of this study is to define clinical and pathologic features associated with poor prognosis after EREC using a large tumor registry. We hypothesize that clinical and pathologic features could be used to accurately discriminate poor survival after EREC.

## Patients and methods

### Data source

A retrospective cohort study was performed using the National Cancer Database (NCDB). The NCDB is a joint program of the Commission on Cancer (CoC) of the American College of Surgeons (ACS) and the American Cancer society. This is a nationwide database containing de-identified oncologic outcomes from more than 1500 commissioned accredited cancer programs in the USA and Puerto Rico. This database captures nearly 70% of all newly diagnosed cancer. ACS and CoC have not verified and are not responsible for statistical validity of the data analysis nor the conclusion derived by investigators. Definitions of database variables are available from the NCDB Participant User Data File data dictionary [10].

### Patient population

We identified patients with esophageal cancer from 2004 to 2019 in the 2020 National Cancer Database. Patients were included in the analysis if they had clinical stage T1aN0M0 cancer (cT1a) and received EREC. Patients were excluded if they had received chemotherapy and/or radiation prior to EREC or surgery after EREC.

### Outcome measure

The primary outcome of interest was overall survival. The 2020 NCDB does not include follow up data on patients diagnosed in 2019, therefore the survival analysis was done for patients from 2004 to 2019.

### Statistical analysis

Patient demographic characteristics included in the analysis were age, sex, Charlson-Deyo comorbidity score/index (CCI), and tumor characteristics. Pathologic features include histology, tumor grade, and lymph node and/or vascular invasion status. Demographic and characteristics were evaluated for their effect on overall survival using univariate cox proportional hazard analysis. Missing data were treated as separate categorical variables (‘unknown’). These were retained in multivariable analysis to preserve sample size and minimize selection bias. To reduce confounding, factors associated with overall survival were included in a multivariable cox proportional hazard analysis. Further survival analysis was conducted using NOMOCOX, a program for generating nomograms for predictive Cox regression models within Stata [11]. The model output was used to generate a risk score. Survival analysis between the risk score strata was performed using Kaplan Meier survival analysis to compare the difference in overall survival. Internal validation of the risk prediction model was performed using 1,000 bootstrap replications to estimate the optimism-corrected concordance index (c-index), providing a measure of the model’s discriminatory ability while adjusting for potential overfitting. Statistical analysis was performed using STATA MP (Version 17.0, College Station, TX).

## Results

The description of the 2169 patients included in the study cohort is shown in Table 1. The average age was 68.4 years old (median: 69 IQR 62–75). Following EREC treatment, pathology was determined to be pT1a in 72.78% of cases, 4.45% of samples classified as pT1b, and less than 0.70% of samples pT2 or pT3. Of pathologic specimens, 15.93% were categorized as well-differentiated, 28.30% were moderately differentiated, 7.60% were poorly differentiated, and the remainder unknown or undetermined. Removed cancers were margin negative in 63.69% of patients and LVI was present in 3.39%. In univariate Cox regression analysis, age, CCI, tumor grade, tumor margin, LVI, tumor histology, and pathologic T-stage were associated with survival (Table 2).

**Table 1:** Clinical and demographic characteristics of patients with esophageal cancer after endoscopic resection stratified by receipt of adjuvant treatment after resection in the 2020 National Cancer Database

Variables	Frequency(%) or Median (IQR)	Percent (%)
Age		
25%	62	
50% (Median)	69	
75%	75	
Mean	68.38	
Sex at birth		
Male	1773	82.12
Female	386	17.88
Charlson-Deyo cormorbidity index		
0	1552	71.89
1	360	16.67
2	151	6.99
> = 3	96	4.45
Tumor grade		
Well-differentiated	344	15.93
Moderately differentiated	611	28.30
Poorly differentiated	164	7.60
Undifferentiated	*	*
Unknown	1033	47.85
Margins status		
Negative margins	1375	63.69
Positive margins	273	12.64
Unknown	511	23.67
Lymphovascular invasion		
No	1141	52.92
Yes	73	3.39
Unknown	942	43.69
Histologic type		
Adenocarcinoma	1929	89.35
Squamous cell carcinoma	127	5.88
Other	103	4.77
Pathologic tumor stage		
pT0	17	1.13
pT1	34	2.26
pT1a	1096	72.78
pT1b	67	4.45
pT2	10	0.66
pT3	*	*
pTX	263	17.46
pTis	17	1.13

Data presented as median (IQR) or n (%) and compared using rank-sum test of chi-square as appropriate. National Cancer Database data use agreement requires suppressing data with fewer than 10 patients (*)

**Table 2:** Univariate Cox analysis of factors associated with overall survival with esophageal adenocarcinoma after endoscopic resection in the 2020 National Cancer Database

Variables	Hazard ratio	p-value	Lower 95% CI	Upper 95% Ci
Age groups				
18–59	ref			
60–69	1.51	0.003	1.15	1.99
70–79	2.83	< 0.001	2.13	3.75
80–90	5.45	< 0.001	4.10	7.25
Charlson-Deyo Comorbidity Index				
0	ref			
1	1.40	0.006	1.10	1.77
2	1.85	< 0.001	1.36	2.51
> = 3	2.05	< 0.001	1.42	2.95
Tumor grade				
Well-differentiated	ref			
Moderately differentiated	1.39	0.021	1.05	1.83
Poorly differentiated	1.85	< 0.001	1.35	2.54
Undifferentiated	0.97	0.965	0.25	3.70
Grade cannot be assessed/ unknown	1.04	0.809	0.78	1.37
Margins				
Negative margin	ref			
Positive margin	1.60	< 0.001	1.29	1.98
Unknown	1.17	0.109	0.97	1.41
Lymph vascular invasion				
Not present	ref			
Present	2.18	0.001	1.38	3.44
Unknown	1.20	0.054	1.00	1.45
Histology				
Adenocarcinoma	ref			
Squamous cell carcinoma	1.63	0.001	1.21	2.20
Other	1.11	0.555	0.79	1.55
Pathologic T-stage				
< = pT1a	ref			
> = pT1b	2.81	< 0.001	1.88	4.21
Unknown	1.15	0.311	0.88	1.52

### Multivariate survival analysis

To reduce confounding, we performed a multivariable Cox hazard analysis of factors associated with overall survival (Table 3). In that analysis, older age, CCI, tumor grade, squamous histology, and > = pT1b T-stage were associated with inferior survival. LVI and positive margin were not associated with survival when adjusted for other variables. To simplify the model, we performed the further analysis pathologic factors associated with tumor. In this model (Table 4), pathologic T1b or greater was associated with inferior survival (HR 2.07, CI 1.33–3.23). These factors were used to generate a survival risk score. The risk score is shown in Table 5, and included grade, margin status, lymphovascular invasion, squamous histology, and high pathologic T-stage.

**Table 3:** Multivariable Cox hazard analysis of factors associated with overall survival among patients with cT1a esophageal cancer after endoscopic resection in the 2020 National Cancer Database

Variables	Haz. ratio	p-value	Lower 95% CI	Upper 95% CI
Age groups				
18–59	ref			
60–69	1.42	0.036	1.02	1.97
70–79	2.58	< 0.001	1.82	3.66
80–90	4.85	< 0.001	3.46	6.78
Charlson-Deyo comorbidity index				
0	ref			
1	1.34	0.008	1.08	1.67
2	1.65	0.022	1.07	2.52
> = 3	1.96	0.004	1.24	3.10
Tumor grade				
Well-differentiated	ref			
Moderately differentiated	1.49	0.032	1.03	2.15
Poorly differentiated	1.41	0.094	0.94	2.12
Undifferentiated	1.15	0.765	0.46	2.88
Grade cannot be assess	1.07	0.728	0.74	1.53
Margin				
Negative margin	ref			
Positive margin	1.12	0.413	0.85	1.48
Unknown	1.09	0.543	0.82	1.45
Lymph vascular invasion				
Not present	ref			
Present	1.27	0.386	0.74	2.20
Unknown	1.10	0.454	0.86	1.41
Histology group				
Adenocarcinoma	ref			
Squamous cell carcinoma	1.64	0.013	1.11	2.42
Other	1.23	0.363	0.79	1.94
pT-stage				
< = pT1a	ref			
> = pT1b	1.89	0.005	1.21	2.94
Unknown	1.13	0.403	0.85	1.49

**Table 4:** Multivariable cox hazard analysis of pathologic tumor factors associated with overall survival among patients with cT1a esophageal cancer after endoscopic resection removing variables where p > 0.100 and rerunning the analysis

Variable	Haz. ratio	p-value	Lower 95% CI	Upper 95% CI
Grade				
Moderate/poorly differentiated	1.29	0.058	0.99	1.69
Well-differentiated or undifferentiated	ref			
Margin status				
Margin negative	ref			
Margin positive	1.31	0.085	0.96	1.69
Lymphovascular invasion				
Not present	ref			
Present	1.40	0.400	0.80	2.46
Unknown	0.97	0.140	0.73	1.27
Squamous cell carcinoma				
No	ref			
Yes	2.04	0.424	1.36	3.07
Pathologic tumor stage				
< = pT1a	ref			
> = pTb	2.07	0.001	1.33	3.23

**Table 5:** Risk score model of pathologic tumor factors amongst patients with cT1aN0M0 esophageal cancer after endoscopic resection in the 2020 National Cancer Database

Risk score item	Category	Points
Grade	Moderate / poorly differentiated / undifferentiated	10
	Well-differentiated or unknown	0
Margin status	Positive	4
	Negative	0
Lymphovascular invasion	Absent or unknown	0
	Present	5
Histology	Squamous	10
	Non-squamous	0
Pathologic T-stage	< = pT1a	0
	> = pT1b	10

### Performance of risk score

**Fig. 1:**
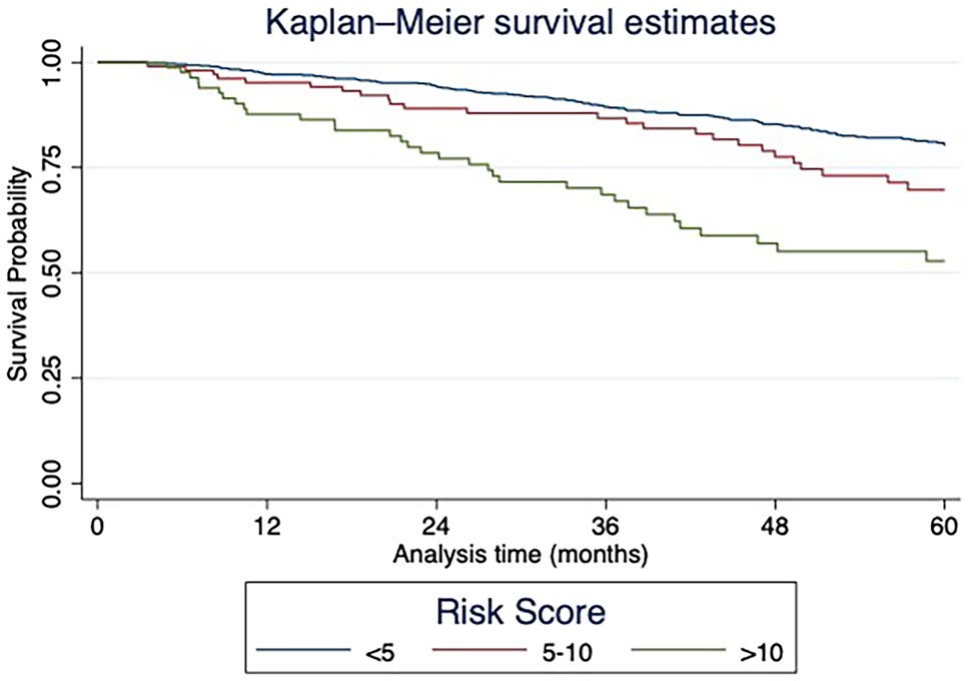
Kaplan Meier survival estimate stratified by risk score among patients with cT1a esophageal cancer after endoscopic resection in the 2020 National Cancer Database

The risk score was analyzed as a continuous variable in a Cox hazard analysis, and increasing score was associated with increased risk of death (HR 1.07 (95% CI 1.05–1.09)). The score was also divided into categorical groups of low (< 5), intermediate (5–10), and high (> 10). The score categorical groups were associated with inferior survival in Kaplan Meier analysis (Log rank test: p < 0.001. Figure 1) and Cox hazard analysis (relative to low: intermediate HR 1.67 (95% CI 1.16–2.42), high HR 3.04 (95% CI 2.15–4.30)). Internal validation was performed using 1,000 bootstrap replications to assess the robustness of the model and correct for overfitting. The optimism-corrected concordance index (c-index) was 0.68, indicating moderate discrimination and acceptable internal validity of the survival prediction model.

## Discussion

To our knowledge, this is the first multi-institutional study to evaluate pathologic factors associated with poor survival after EREC of cT1a esophageal cancer. While EREC has been lauded as a safe and effective method of treating early esophageal cancer, some patients will experience recurrence after resection. Even after successful EET of T1 EAC, neoplastic recurrence was seen in 8.3% of cases after 5 years in prior studies [12]. We believe that the risk scores defined in this study can be used to delineate which patients are at highest risk for death. These data are invaluable for patient counseling and can be used to identify patients who may benefit from additional treatment, such as chemotherapy or radiation.

While several smaller studies have looked at risk factors for poor prognosis after endoscopic treatment of esophageal cancer, much of these data are extrapolated from surgical patients [13]. In these studies, occult lymph node metastasis was the outcome of interest as these would not be diagnosed by a purely endoscopic therapeutic approach and would represent a patient who received inadequate treatment by endoscopic therapy alone. In a study of 160 consecutive esophagectomy patients at a single institution, one study suggested that nodal metastasis was more common in tumors with submucosal invasion, a non-flat shape and lymphatic invasion [14]. Tumor depth and macroscopic shape of the tumor were independent risk factors for lymph node metastases [14].

An analysis of the NCDB identified infiltration of submucosa, tumor size exceeding 10 mm, and poor tumor differentiation as factors that were independently associated with risk of nodal disease with lymph node metastasis only present in 4.8% of patients without these risk factors [15]. This study adds to this analysis by focusing on patients who were treated endoscopically and adding additional relevant variables, including positive margin. In contrast to the other studies, we found that LVI was not associated with increased risk of death, which may be due to the large number of patients for which this was not documented in our study [15]. In our study LVI status was listed as unknown in 43% of cases. Additionally, existing research supports the presence of LVI as a risk factor for nodal metastasis and/or disease progression following surgical or endoscopic treatment. While our findings are concordant with existing literature regarding the importance of histology and tumor grade in the progression of carcinoma, following treatment (surgical or endoscopic), the current existing literature does not fully evaluate the risk factors for carcinoma recurrence and disease progression in patients undergoing EREC.

The question of how patients with high-risk features such as LVI or a positive deep margin should be treated following initial endoscopic treatment is outside the scope of this study, but we believe that the results of this study could be used to identify potential candidates for additional treatment. Certainly, we believe that surgical candidates should be considered for esophagectomy, which is likely associated with best oncologic outcomes for many high-risk patients. Current NCCN guidelines recommend EREC followed by ablation as the primary mode of treatment for stage pT1a SCC with esophagectomy as the second line option in patients who are medically able to undergo the procedure. These guidelines recommend similar treatment for patients with adenocarcinoma of stage pTis, pT1a or pT1b, N0 who are able to undergo treatment [2]. The NCCN recommends that decisions regarding adjuvant treatment in the form of preoperative chemoradiation, perioperative chemotherapy, post operative therapy and definitive chemoradiation must be developed with a health care team with experience in the use of anticancer agents and management of toxicities with best management of any cancer patient being inclusion in a clinical trial [2].

By excluding patients who underwent esophagectomy after EREC, we intentionally focused our analysis on those managed definitively with endoscopic therapy. However, this approach may have excluded patients with high-risk pathologic features who were appropriately triaged to surgical resection based on their pathology. As a result, our findings may underestimate the prognostic impact of adverse features such as deep invasion or LVI, and the risk score derived here may be less applicable to the full spectrum of early-stage esophageal cancer patients encountered in clinical practice. Future studies including all treatment pathways may help clarify the broader prognostic significance of these features. However, this risk score may help identify patients at higher risk of poor survival after EREC. Clinicians may consider these risk categories in shared decision-making, particularly when evaluating patients for adjuvant therapy or for more aggressive surveillance. For instance, patients with a high-risk score may be appropriate candidates for multidisciplinary review and discussions about esophagectomy or chemoradiation, even if technically resectable disease was removed endoscopically.

There are several limitations to this study. The NCDB contains de-identified data, and the accuracy of the data entries cannot be adjudicated. There may also be variation in the skill of the endoscopist and/or pathologist which could influence the results of this study. Another limitation is that we have not adjusted for the adjuvant treatment that each patient received. This may bias the findings of our study if providers were offering more aggressive treatment based on specific findings (such as positive margin or LVI). We also were unable to determine in patients with EAC, if they had undergone successful ablation therapy to achieve complete remission of intestinal metaplasia or complete remission of dysplasia. The NCDB does not include data on recurrence type, location, or disease-free survival, limiting our ability to assess recurrence patterns or time to recurrence in this cohort. The NCDB also does not include granular data on diagnostic imaging or staging modalities (e.g., EUS or PET/CT), which may impact the accuracy of clinical staging and subsequent treatment planning. The NCDB does not document whether patients underwent ablation following EREC, which is a common adjunctive treatment in Barrett’s-associated early adenocarcinoma. This limits our ability to assess the impact of ablation on outcomes. While our model demonstrated moderate discriminatory ability on internal validation, external validation using an independent dataset is necessary to confirm its generalizability and clinical utility. Future studies should consider validation in large, prospectively maintained registries or institutional databases with detailed clinical annotation and recurrence data to further assess performance and applicability in diverse patient populations.

## Conclusion

Many patients cannot undergo esophagectomy due to comorbidities, age and lifestyle concerns. For those patients we believe that this analysis could be used to identify patients for adjuvant treatments. This study suggests that increased age, Charlson-Deyo Comorbidity Index, undifferentiated grade, histology, positive margin status, and pathologic T-stage > = pT1b are independently associated with inferior overall survival following EREC. This risk score may serve as a tool to stratify patients after EREC, guide clinical decisions regarding surveillance and adjuvant therapy, and support individualized treatment planning.
